# Death to weak PowerPoint: strategies to create effective visual presentations

**DOI:** 10.3389/fpsyg.2014.01138

**Published:** 2014-10-08

**Authors:** Rodney M. Schmaltz, Rickard Enström

**Affiliations:** ^1^Department of Psychology, MacEwan UniversityEdmonton, AB, Canada; ^2^Department of Decision Sciences and Supply Chain Management, School of Business, MacEwan UniversityEdmonton, AB, Canada

**Keywords:** teaching resources, PowerPoint, presentation software, educational media, visual design

## The problem with powerpoint

There is nothing more frustrating than sitting through a presentation bombarded by slide after slide of small text, difficult to read graphs, irrelevant clip art images, and poorly designed templates. Often to blame is the use and abuse of PowerPoint^1^ (e.g., Tufte, 2003; Bumiller, 2010). Academics typically only endure weak PowerPoint presentations at conferences, while university students may be exposed to them several times a day for an entire semester. Strong PowerPoint presentations enhance student engagement and help students retain information (e.g., Susskind, 2005), while weak PowerPoint slides can lead to distraction, boredom, and impeded learning (Savoy et al., 2009).

**Figure 1:**
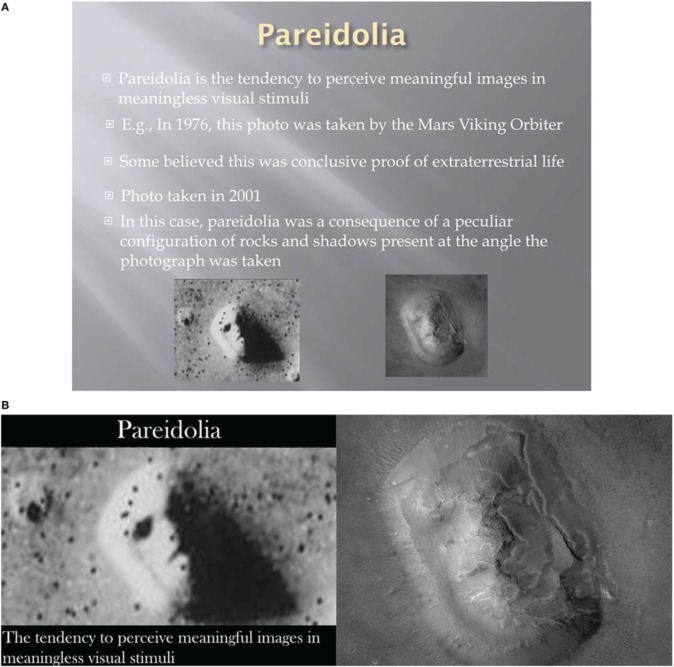
**(A)** A poorly designed slide. This slide relies on text-heavy bullets. The photos are too small, and the text does not stand out against the background. **(B)** Another approach to presenting the information in **(A)**. Unnecessary text is removed, the photos are enlarged, and the content is broken down into two separate slides. The instructor should discuss the material in the bullet points on the slide in **(A)**, but it need not be printed on the slide.

The authors of this paper became interested in improving their PowerPoint slides after observing several presentations that badly misused PowerPoint, and realizing that they made many of the same mistakes. Our slides used standard, boring templates; were text heavy, and included grainy gif images—embarrassingly, some of which were even animated. For example, Figure 1A contains a slide that was prepared for a lecture in an introductory psychology course. The slide uses a template that makes the text difficult to read, there are several lengthy bullets, and the photos are too small. To make matters worse, the instructor had the slide heavily animated—bullet points flew in, swirled around, and even made sound. Needless to say, students were not impressed.

Outstanding PowerPoint slides will not save a weak lecture; however, weak PowerPoint will certainly diminish a strong one. The purpose of this brief article is to provide some basic suggestions and resources for instructors who wish to improve their PowerPoint presentations, and in doing so, create more engaging and informative lectures for students.

## The basics of powerpoint

For the most part, graduate school does not train instructors how to properly use presentation software. Excluding basic slides provided by publishing companies, instructors are given little guidance on what a PowerPoint presentation should look like. The software itself does not provide any assistance. Upon opening PowerPoint, users are presented with standard templates with space to add a title at the top of the slide, and a block of text to add bullet points. For the most part, this has been shown to be an ineffective means to convey information (Garner and Alley, 2011). Whenever possible, we recommend avoiding the use of templates that are included with PowerPoint. The images on the templates are tired, as templates with images that look the best tend to be the ones that are used the most.

Creating a well-designed PowerPoint presentation is not intuitive (Kosslyn et al., 2012), so where should an instructor look for guidance? Fortunately, there are many excellent resources. Books such as Presentation Zen (Reynolds, 2012), Slide:ology (Duarte, 2008), and Presentation Secrets (Kapterev, 2011) focus on design, and provide a different way of approaching PowerPoint. From these resources, and the literature on design, educational and cognitive psychology, we can find some basic points that should be considered when using PowerPoint for lectures.

### Font and text

Beyond aesthetics, font choice has an impact on how students process information. For example, Song and Schwarz (2008) presented students with instructions for an exercise routine that was printed in either an easy-to-read font, or one that was difficult to read. The researchers found that students who read the instructions in a difficult-to-read font were less willing to make the exercise a part of their daily routine, and perceived the exercise as requiring more time to complete than those students who read the same instructions in a clear font. Other researchers have found a similar impact with difficult-to-read fonts (e.g., Schwarz, 2004; Rhodes and Castel, 2008; Sanchez and Jaeger, 2014). A poorly chosen font can negatively impact the perception of the material, and the perception of the presenter themselves (Oppenheimer and Frank, 2008). Even an announcement as monumental as the discovery of the Higgs Boson can be marred by a bad font. Comic Sans was used in the presentation of the discovery, and received mockery in the press (e.g., Urquhart, 2011). Font choice may seem inconsequential, but can have a major impact on a presentation.

Presenters should be wary of the colors used for presenting text and graphs. Color-blindness is not uncommon. For this reason, slides should not have color schemes with red on green or blue on yellow. For the most part, we recommend using either white on black, or black on white. While having white text on a black background can lead to some bleeding of the text, it has been our experience that students are still able to see the material clearly. This is not to say that all slides should be simply black and white. Text boxes can be placed over graphics and images, allowing for variety in the slides, and clarity in the text (see Figure 1B). While the research on the ideal font is mixed (e.g., Duarte, 2008), Mackiewicz (2007) recommends Gill Sans as a safe choice.

### Animations

When PowerPoint was first available, there was a certain novelty to having text fly onto the screen, spin around, burst into flames, and fly out. The reality now is that students are used to these effects, and they are more distracting than anything else. There are times when animations are necessary, such as when an instructor may not want all of the text on a slide to be available at once to students. While it may be useful for separate points to appear on the screen at different times, there should be no distracting animations (Daffner, 2003).

### Videos and images

The choice of images or graphics is important. Verbal information supplemented with appropriate images is better retained than information presented simultaneously with both graphics and text (Mayer, 2009). This means that students remember more if instructors speak to images on a slide, rather than images *and* redundant text (i.e., bullet points that reiterate what the speaker is discussing). That said, images and graphics must be chosen carefully. The images used on a slide must be consistent with the message of the presenter. Images that are superfluous or inconsistent with the instructor's verbal output may actually hinder student retention (e.g., Bartsch and Cobern, 2003). Instructors should also be sure to avoid low-resolution images (i.e., no less than 1600 × 1200 pixels), clipart, or images with watermarks. A grainy image with a watermark is distracting and may come across as unprofessional. The same is true of clip art. Including clip art in a presentation is somewhat dated, and again, is often viewed as unprofessional (Alley, 2013). Fortunately, there are a number of resources available to find suitable images^2^.

Videos should be embedded in presentations. Relying on an internet connection to stream content can be risky and break up the flow of the presentation. There are numerous sites available to legally download video content. Instructors that are concerned with copyright issues should check with the copyright specialist at their institution.

Keeping the above points in mind, let us revisit Figure 1A. A better approach to this slide is to remove unnecessary text, enlarge the images, and break the single slide down into two separate slides, as we see in Figure 1B. With this approach, the instructor can place the focus on the images, which is critical in this example, and speak to what the students are viewing. This is far more engaging than reading the bullet points off of the slide in Figure 1A.

## The student experience

In our experience, students need some preparation to deal with PowerPoint presentations that are not loaded with text. As students have become used to seeing text-heavy slides, many have gotten into the routine of simply writing down everything that is on the slide and moving on. With less text on slides, and graphics that enhance the key points of the instructor, students need to pay attention to what is actually being said in the classroom. As well, students are not going to be able to write down everything, as some are previously accustomed to. At the beginning of the term, we provide students with an overview of how the slides are going to look also give instruction on how students can become active listeners. Students are taught to listen for key points, minimize the amount of note taking and maximize the amount of attention that is spent on what the instructor is saying. There can certainly be an adjustment period for students. Still, we typically find that attendance goes up, student engagement increases and grades improve.

## Who has the time?

Between writing grants, collecting data, writing papers, and preparing lectures, there is intense pressure placed on instructors. To try to minimize the time spent on preparing lectures, many instructors understandably spend little time creating PowerPoint slides, or simply use slides provided by the publisher. Unfortunately, publisher slides tend to be text heavy, and often do nothing more than regurgitate textbook material. While many instructors wish to improve their slides, there is a common concern that there is simply not enough time to do so. In our experience, properly created slides actually take less time to create than the more commonly used text-heavy slides with a header and several bullets. We encourage instructors to look at their current slides and consider if the images on the slides are complimenting what is being said and if the text is enhancing the lecture. If the answer to either of these questions is negative, the instructor needs to decide whether to stop using PowerPoint—which is not necessarily a bad option, we have both seen brilliant presentations that did not use PowerPoint—or how to change things for the better.

One benefit of updating and enhancing PowerPoint slides is that it forces instructors to think about the content of their course. If there is not an image, or small amount of text that can explain a concept, it could be that the concept does not lend itself to the style of PowerPoint—in that case, PowerPoint should not be used. It might also be that the instructor is not familiar enough with the material to be able to create an appropriate slide. We found that when updating our lectures, topics that we were comfortable with were easy to present with minimal text and an appropriate image. This was more challenging for topics that we were less comfortable with. This forced us to review our course material, and ultimately, improve the quality of the lectures.

As previously mentioned, many instructors use the slides provided by publishing companies. While we argue these slides don't stand up well on their own, they still have value. The publisher's slides contain the basics from the textbook, and may be useful as a way to frame lectures. These slides could be considered an outline of what may be covered, and then altered to reduce text, add meaningful images or graphs, and supplemented with relevant examples from the literature.

## Final thoughts

Student engagement can be difficult, as students have more temptation than ever to tune out from a dull lecture. Laptops, phones and tablets all contain the lure of social media, surfing the web, text messaging, or simply reading a magazine or book, and this can all be done under the guise of listening to a lecturer. Effective PowerPoint presentations can prevent student distraction and facilitate a better student experience. There is no need for students to endure substandard presentations, and as such, we challenge all instructors to put weak PowerPoint to rest.

### Conflict of interest statement

The authors declare that the research was conducted in the absence of any commercial or financial relationships that could be construed as a potential conflict of interest.

## Footnotes

^1^Note that in this article we are using the denotation PowerPoint to refer to any presentation software (e.g., Keynote, Impress, etc.).

^2^Google images offers a wide selection of free images, though there may be copyright issues. Other valuable online resources include Getty Images, iStock Photo, Flickr Creative Commons, and Everystockphoto.
